# Growth of a human mammary tumor cell line is blocked by galangin, a naturally occurring bioflavonoid, and is accompanied by down-regulation of cyclins D3, E, and A

**DOI:** 10.1186/bcr1391

**Published:** 2006-03-27

**Authors:** Tessa J Murray, Xinhai Yang, David H Sherr

**Affiliations:** 1 Department of Environmental Health, Boston University School of Public Health, Boston, MA, USA

## Abstract

**Introduction:**

This study was designed to determine if and how a non-toxic, naturally occurring bioflavonoid, galangin, affects proliferation of human mammary tumor cells. Our previous studies demonstrated that, in other cell types, galangin is a potent inhibitor of the aryl hydrocarbon receptor (AhR), an environmental carcinogen-responsive transcription factor implicated in mammary tumor initiation and growth control. Because some current breast cancer therapeutics are ineffective in estrogen receptor (ER) negative tumors and since the AhR may be involved in breast cancer proliferation, the effects of galangin on the proliferation of an ER^-^, AhR^high ^line, Hs578T, were studied.

**Methods:**

AhR expression and function in the presence or absence of galangin, a second AhR inhibitor, α-naphthoflavone (α-NF), an AhR agonist, indole-3-carbinol, and a transfected AhR repressor-encoding plasmid (*FhAhRR*) were studied in Hs578T cells by western blotting for nuclear (for instance, constitutively activated) AhR and by transfection of an AhR-driven reporter construct, *pGudLuc*. The effects of these agents on cell proliferation were studied by ^3^H-thymidine incorporation and by flow cytometry. The effects on cyclins implicated in mammary tumorigenesis were evaluated by western blotting.

**Results:**

Hs578T cells were shown to express high levels of constitutively active AhR. Constitutive and environmental chemical-induced AhR activity was profoundly suppressed by galangin as was cell proliferation. However, the failure of α-NF or *FhAhRR *transfection to block proliferation indicated that galangin-mediated AhR inhibition was either insufficient or unrelated to its ability to significantly block cell proliferation at therapeutically relevant doses (IC_50 _= 11 μM). Galangin inhibited transition of cells from the G_0_/G_1 _to the S phases of cell growth, likely through the nearly total elimination of cyclin D3. Expression of cyclins A and E was also suppressed.

**Conclusion:**

Galangin is a strong inhibitor of Hs578T cell proliferation that likely mediates this effect through a relatively unique mechanism, suppression of cyclin D3, and not through the AhR. The results suggest that this non-toxic bioflavonoid may be useful as a chemotherapeutic, particularly in combination with agents that target other components of the tumor cell cycle and in situations where estrogen receptor-specific therapeutics are ineffective.

## Introduction

Flavonoids are a diverse class of naturally occurring polyphenolic plant compounds that have a variety of therapeutically important biological activities. Several thousand plant flavonoids have been identified and biologically significant levels of bioflavonoids are consumed (about 1 g/day) by humans living in Western cultures [1]. Generally considered to be non-toxic [2], flavonoids have been touted as anti-inflammatory, anti-oxidant, chemopreventatives with the potential to be used for prevention or treatment of such diverse diseases as arthritis [3], cardiovascular disease [4], and several cancers, including breast cancer [5,6].

Galangin (3,5,7-trihydroxyflavone) belongs to one class of flavonoids known as flavonols. It comprises approximately 10% of the ethanol extract from *Alpinia officinarum*, a plant used for many years in Asia as an herbal therapeutic [7], and is a major component of propolis, an anti-inflammatory composite gum produced by honeybees [8-10]. Among other biological activities, galangin blocks *iNOS *mRNA induction during inflammatory responses [11], down-regulates Cox-2 transcription [12], inhibits viral replication *in vitro *[13], and suppresses bacterial cell growth [14]. Recently, we generated data in a murine model of B lymphocyte development that suggest that galangin may further affect cellular biology through its interaction with an important receptor/transcription factor, the aryl hydrocarbon receptor (AhR) [15], implicated in spontaneous and carcinogen-induced mammary tumorigenesis [16-18].

The AhR is a member of the Per/ARNT/Sim (PAS) family of transcription factors. PAS proteins have been shown to contribute to angiogenesis, neurological development, hypoxia responses, and circadian cycle [19-22]. The AhR is best known for its responsiveness to environmentally ubiquitous carcinogens such as polycyclic aromatic hydrocarbons (PAHs), dioxins (for example, 2,3,7,8 tetrachlorodibenzo-*p*-dioxin/TCDD), and polychlorinated biphenyls [23-25]. Once activated by these lipophilic pollutants, the AhR translocates to the nucleus where it binds to a second member of the PAS family, the aryl hydrocarbon nuclear translocator (ARNT), and to transcriptional co-activators or co-repressors [26-30]. The activated AhR complex then binds specific DNA recognition sequences and modulates transcription of a variety of genes [27,31,32]. Significantly, AhR activation can be blocked with galangin [15,33].

The most thoroughly characterized outcome of AhR activation is the induction of *CYP1 *genes encoding the cytochrome P-4501A1, 1A2, and 1B1 monooxygenases. These enzymes metabolize parent PAHs and polychlorinated biphenyls into mutagenic intermediates [34-39]. However, it is now becoming clear that the AhR, a highly conserved protein, plays an important role in cell cycle and apoptosis regulation in the absence of environmental chemicals [40-49]. Indeed, the potential role of the AhR in tumorigenesis has inspired the possible use of AhR modulators for breast cancer therapy [50].

Because it is an AhR inhibitor [15], it seemed plausible that galangin has the potential to block formation of mutagenic metabolites in tumor cells and to regulate human mammary cancer cell proliferation. The former possibility is supported by the demonstration that galangin blocks *CYP1A1 *induction and DNA-adduct formation in a human mammary tumor cell line [51]. The latter possibility, that galangin can alter human tumor cell proliferation after the transformation process has begun, was investigated herein. Particular emphasis was placed on the possibility that galangin mediates its presumptive growth regulatory effects through constitutively active tumor cell AhR and on the influence that galangin may have in altering levels of cyclins critical to maintenance of cell growth. Since at least some flavonoids are xenoestrogens [52], and since modulation of estrogen receptors (ERs) would be expected to affect cell proliferation, estrogen receptor modulation was eliminated as a confounder by performing studies on ER^- ^human Hs578T tumor cells. Consequently, the results presented here have a bearing on the potential for galangin to be used as a chemotherapeutic in cases where ER-dependent therapeutics are either contraindicated or not effective because of ER loss.

## Materials and methods

### Reagents

DMEM, RPMI, calcium- and magnesium-free PBS, L-glutamine, penicillin/streptomycin, trypsin-EDTA, and heat inactivated FCS were supplied by Invitrogen (Carlsbad, CA, USA). Galangin, indole-3-carbinol (I3C), and α-naphthoflavone (α-NF) were purchased from Aldrich Chemical Co. (Milwaukee, WI, USA) and dissolved in dimethylsulfoxide (99.9% high-performance liquid chromatography grade; Sigma Chemical Co., St Louis, MO, USA) at concentrations that were 1,000-fold higher than the desired final concentration. Insulin was obtained from Sigma.

### Cell culture

The ER^- ^Hs578T human breast cancer epithelial cell line was purchased from the American Type Culture Collection (Manassas, VA, USA) and grown in DMEM (Sigma) supplemented with 10% FCS, 10 μg/ml insulin, 50 u/ml penicillin, 50 u/ml streptomycin, and 2 mM L-glutamine. Cells were maintained at subconfluency at 37°C in humidified air containing 10% CO_2 _by splitting cultures 1:4 every 3 to 4 days.

### [^3^H]-Thymidine incorporation

Log phase Hs578T cells (10^3^/well) were plated into 96-well tissue culture plates and allowed to adhere overnight. Cells were incubated with 1 μCi ^3^H-thymidine/well (NEN Life Science Products, Boston, MA, USA) in triplicate wells and dosed with 0.1% vehicle, 10^-4 ^to 10^-6 ^M galangin, I3C, or α-NF for 18 h. Cells were harvested onto filter mats using a PHD cell harvester (Brandel, Gaithersburg, MD, USA). ^3^H-thymidine retained on the filter was detected using a scintillation counter (Becton/Dickinson, San Jose, CA, USA). Triplicates for each data point in each experiment were averaged to give an 'n' of one.

### Transient transfections and reporter assays

The *Fundulus heteroclitus AhRR *expression vector [53] was generously provided by Dr M Hahn and Dr S Karchner (Woods Hole Oceanographic Institution). We and others have shown that this construct is a potent inhibitor of both human and murine AhR activity [53,54]. The *pGudLuc6.1*-firefly luciferase reporter construct (*pGudLuc*) was kindly provided by Dr M Denison (UC Davis). AhR-dependent expression of this reporter is driven by four aryl hydrocarbon response elements (AhREs) derived from the *CYP1A1 *promoter [55].

Hs578T cells (10^5^/well) were seeded into 6-well culture plates and grown to 70% to 80% confluence. Lipofectamine 2000 transfection reagent (Invitrogen) was used according to the manufacturer's instructions to transfect cells. The renilla luciferase vector *phRL-TK *(0.5 μg/well) was co-transfected with the 0.1 μg control vector (*pGL3*) or with *pGudLuc *per well. Where indicated, 0.125 to 0.5 μg of *pcDNA-FhAhRR *or control *pcDNA *were added with or without the reporter construct to the transfection mixture. For each experiment, the amount of total DNA transfected was equilibrated with parental expression vectors. Cells were incubated for 18 hours, washed twice with PBS (pH 7.2), and resuspended in 75 μl RPMI prior to luciferase assays. Luciferase activity was determined with the Dual Glo Luciferase system (Promega, Madison, WI, USA), allowing sequential reading of the firefly and renilla signals. Briefly, cells were lysed in equal volumes of cell lysis buffer (Promega) and RPMI for 20 minutes, transferred to a 96-well white wall plate, and analyzed using a Reporter Luminometer (Promega). The renilla signal was read after quenching the firefly output, thus allowing normalization between sample wells.

For experiments in which cell proliferation was assayed after *FhAhRR *or control *pcDNA *transfection, transfected cells were resuspended in RPMI and plated (10^3 ^cells/well) into 96-well tissue culture plates in triplicate and allowed to adhere overnight. Cells were incubated with 1 μCi ^3^H-thymidine/well (NEN Life Science Products) and triplicate wells assayed for ^3^H-thymidine incorporation as described above. Results in the triplicates were averaged for each data point in each experiment.

### Cell cycle and apoptosis analyses by flow cytometry

Hs578T cells (10^5^/well) were seeded into 6-well tissue culture plates and allowed to adhere overnight. Growth arrest was achieved by washing the cells three times with cold PBS before adding supplemented DMEM containing no FCS. In preliminary experiments it was determined that 48 hours without FCS was required to arrest 80% to 90% of the cells in G_0_/G_1_. Less than 5% of these cells stained with trypan blue, indicating a high level of viability. Cells were rescued from growth arrest by adding FCS to culture wells (10% final concentration) with 0.1% vehicle, galangin, I3C, or α-NF. Cells were harvested 24 hours later and analyzed for cell cycle as described below.

Cell cycle analyses and apoptosis quantification were performed by staining cellular DNA with propidium iodide (PI) in permeabilized cells as we have previously described [56-58]. Cells were trypsinized, pelleted, and washed in cold PBS containing 5% fetal bovine serum and 0.02 M sodium azide. Cells were centrifuged for 5 minutes at 1,000 rpm at 4°C and resuspended in 0.3 ml hypotonic buffer containing 50 μg/ml PI (Sigma), 1% sodium citrate, and 0.1% Triton X-100 and stored protected from light until analysis. Flow cytometry was performed on a Becton/Dickinson FACScan flow cytometer. Data (5,000 events) were collected on both linear and log scales to assess cell cycle and apoptosis, respectively.

### Western immunoblotting

Cells were scraped into cold PBS and resuspended in P_10_EG lysis buffer containing 10 mM sodium phosphate, 0.75 mM EDTA, 10% glycerol, 0.125% Triton X-100 and 1.0% protease inhibitor cocktail (Sigma). Cells were lysed after 50 strokes in a Dounce tissue homogenizer (Bellco Glass, Vineland, NJ, USA) and lysis was confirmed by light microscopy. After 15 minutes on ice, cells were centrifuged for 15 minutes at 13,000 rpm at 4°C. Supernatants were removed and stored at -80°C until western analysis. Proteins (30 μg/sample) were electrophoresed through 10% SDS-polyacrylamide gels for 1.5 hours at 100 V. Proteins then were transferred to nitrocellulose membranes and membranes blocked for 1 hour at room temperature with 5% non-fat dry milk in PBS with 0.5% Tween-20 (PBS-T). Membranes were probed with the following primary antibodies diluted 1:200 in PBS-T containing 5% non-fat dry milk: rabbit anti-AhR (Santa Cruz Biotechnology, Santa Cruz, CA, USA), mouse anti-cyclin A (BD Pharmingen, San Diego, CA, USA), rabbit anti-cyclin D1, rabbit anti-cyclin D3, or rabbit anti-cyclin E (Santa Cruz Biotechnology). Membranes were washed 4 times for 5 minutes each with PBS-T and incubated for 1 hour at room temperature with goat anti-rabbit IgG horseradish peroxidase conjugate (Bio-Rad, Hercules, CA, USA) or goat anti-mouse IgG horseradish peroxidase conjugate (Sigma) at a dilution of 1:20,000 or 1:5,000, respectively, prepared in PBS-T containing 5% non-fat dry milk. Membranes were washed extensively with PBS-T and developed with enhanced chemiluminescence. Blots were reprobed up to three times with a different primary antibody after treating for 15 minutes with stripping solution (Chemicon, Temecula, CA, USA) and incubating twice for 5 minutes with blocking solution (Chemicon).

### Image analyses

Image analyses were performed on western immunoblotting autoradiographs that were digitally scanned using a Cytocore, Inc. (Chicago, IL, USA) densitometer. To compare relative band densities between immunoblots, all bands were normalized using β-actin band densities.

### Statistical analyses

Statistical analyses were performed with Statview (SAS Institute, Cary, NC, USA). Data from triplicate samples were averaged for each data point for an 'n' of one in each experiment. Data from a minimum of three experiments are presented as means + standard errors (SE). One-factor ANOVAs were used to analyze the data. A Fisher PLSD (protected least significant difference) *post hoc *comparisons test was used to determine significant differences.

## Results

### Galangin represses constitutive and ligand-induced AhR transcriptional activity

The AhR is expressed at high levels in many rapidly growing human and murine tumors [16,33,40-46,50,59,60]. More specifically, high levels of both cytoplasmic and nuclear AhR characterize rodent and human tumors, including mammary tumors induced with an AhR ligand [16,43,59,61]. These and other results [59,62,63] suggest that the AhR is constitutively active in rapidly growing transformed cells. To extend these studies to a human tumor model in which the effects of AhR inhibitors such as galangin can be studied, AhR expression and subcellular localization were determined in a human mammary tumor cell line, Hs578T.

**Figure 1:**
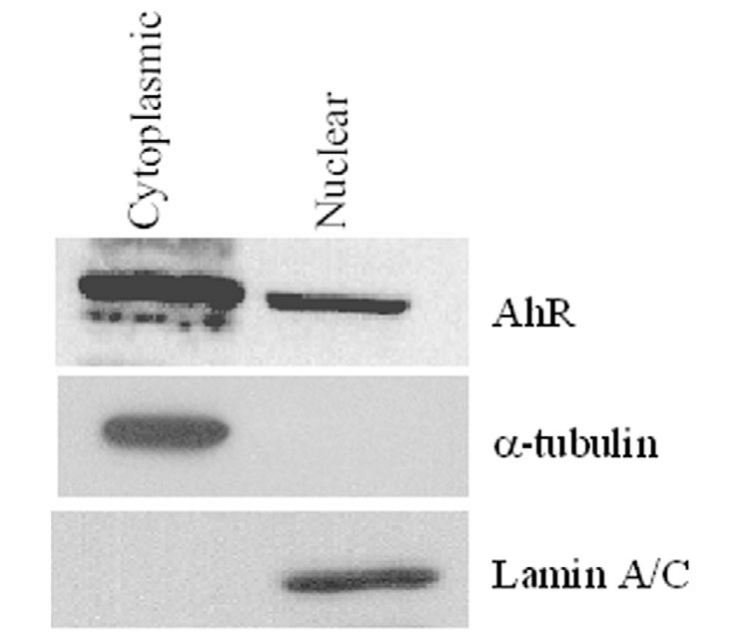
Hs578T cells express nuclear aryl hydrocarbon receptor (AhR). Cytoplasmic and nuclear cell extracts prepared from subconfluent monolayers of malignant, estrogen receptor negative Hs578T cells were analyzed by western immunoblotting with AhR-specific antibody following SDS-PAGE. Blots were stripped and re-probed for lamin A/C and α-tubulin to confirm purity of the nuclear and cytoplasmic cell fractions, respectively. Representative data from a total of three experiments are shown.

As seen with primary tumors *in vivo *[16], significant levels of AhR protein were detected in both the cytoplasm and the nuclei of Hs578T cells (Figure 1), a result consistent with constitutive AhR activity in this line. The presence of the AhR in this cell line is consistent with a previous report from Wang and colleagues [64] in which binding of a functional AhR to its cognate DNA response element was demonstrated after agonist treatment.

If this nuclear AhR were indeed constitutively active, it would be predicted that transient transfection of an AhR-driven luciferase reporter construct (*pGudLuc*) would result in significant levels of background transcriptional activity and that this activity would be inducible with AhR ligands and inhibitable with AhR competitive inhibitors, including galangin. To test these predictions, Hs578T cells were transiently transfected with the renilla luciferase plasmid *phRL-TK *to control for transfection efficiency and with control *pGL3 *plasmid or *pGudLuc *plasmid and treated with vehicle, one of two AhR inhibitors (galangin, α-NF), or an AhR agonist (I3C). Renilla and firefly luciferase activities were assayed 18 hours later.

**Figure 2:**
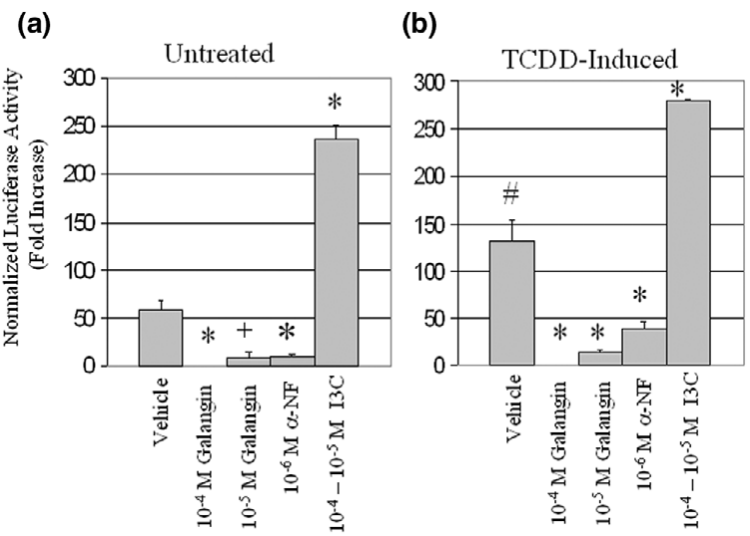
Galangin inhibits aryl hydrocarbon receptor-dependent *pGudLuc *reporter activity. Hs578T cells were left untransfected or were transfected with 0.5 μg/well renilla luciferase vector *phRL-TK *and 0.1 μg control *pGL3 *or *pGudLuc *vector per well and treated with 10^-4 ^to 10^-5 ^M galangin, 10^-4 ^to 10^-5 ^M indole 3-carbinol (I3C), or 10^-6 ^M α-naphthoflavone (α-NF) in the **(a) **absence or **(b) **presence of 10^-9 ^M 2,3,7,8-tetrachlorodibenzo-*p*-dioxin (TCDD). Cells were harvested 18 hours later and luciferase activity assayed. Firefly luciferase activity was normalized to renilla activity in each experiment. **(a) **Data pooled from 4 to 16 experiments are presented as the average fold increase relative to non-transfected cells + standard error. An asterisk (*) indicates a significant difference relative to vehicle-treated controls, *p* < 0.02. A cross (+) indicates *p* = 0.056. **(b) **Data pooled from 4 to 16 experiments are presented as the average fold increase relative to non-transfected cells + standard error. An asterisk (*) indicates a significant difference relative to vehicle-treated controls, *p* < 0.02. A hash sign (#) indicates a significant increase in activity relative to untreated, *pGudLuc*-transfected controls.

As predicted, transfection with *pGudLuc *increased normalized luciferase activity approximately 50-fold relative to *pGL3*-transfected controls in this series of experiments (Figure 2a). Addition of 10^-4 ^M galangin completely blocked the constitutive level of reporter activity (*p* < 0.02). At a lower dose (10^-5 ^M), galangin tended to decrease the activity, although the data did not reach statistical significance in the three experiments performed (*p* = 0.056). A synthetic flavonoid, α-NF (10^-6 ^M), previously shown to block AhR activity [65,66], similarly reduced constitutive *pGudLuc *activity (*p* < 0.02). As expected from previous studies [50], I3C, an AhR agonist, significantly induced *pGudLuc *reporter levels.

A similar profile was seen when AhR activity was induced with 10^-9 ^M TCDD (Figure 2b). That is, TCDD significantly increased the baseline level of *pGudLuc *activity (Figure 2b, first histogram) relative to untreated controls (Figure 2a, first histogram) while 10^-4 ^to 10^-5 ^M galangin or 10^-6 ^M α-NF significantly blocked this induction (*p* < 0.02). I3C, together with TCDD, resulted in the greatest increase in *pGudLuc *activity. These data demonstrate that both galangin and α-NF can suppress constitutive and TCDD-induced, AhR-dependent transcriptional activity in a human mammary tumor cell line.

### Galangin inhibits Hs578T cell proliferation

**Figure 3:**
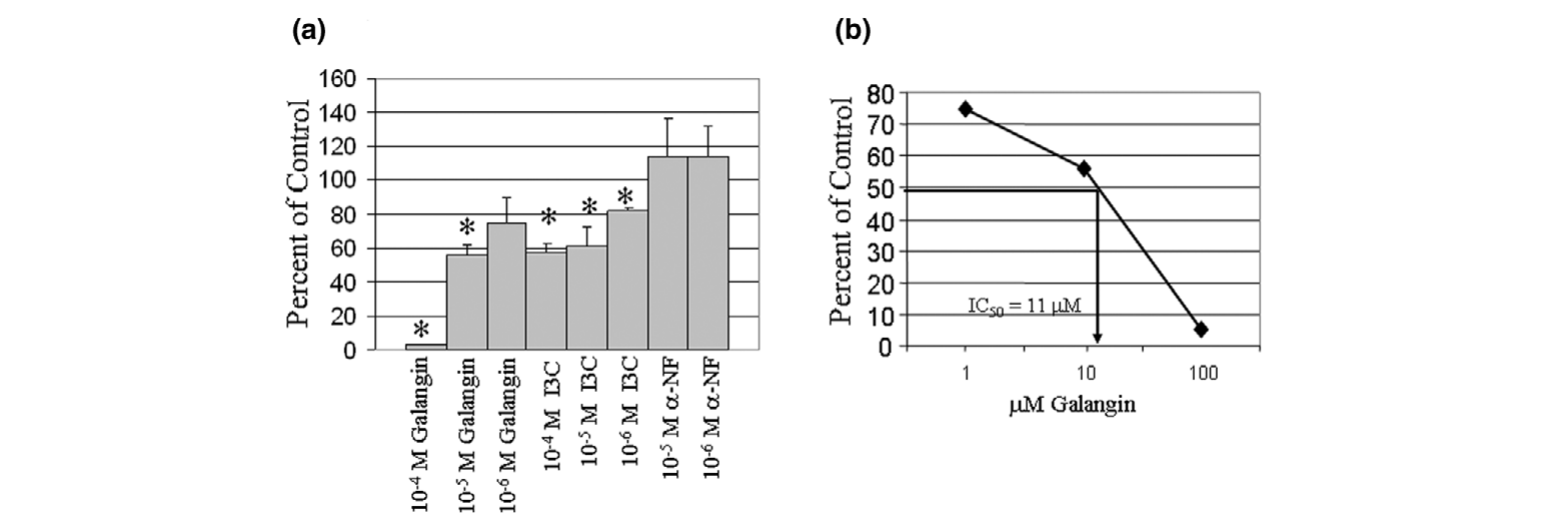
Galangin inhibits proliferation of Hs578T breast cancer cells. Hs578T cells were treated in triplicate with vehicle, 10^-4 ^to 10^-6 ^M galangin, 10^-4 ^to 10^-6 ^M indole 3-carbinol (I3C), or 10^-5^-10^-6 ^M α-naphthoflavone (α-NF) and grown in 3H-thymidine-containing media for 18 hours. Triplicates were averaged for each point in each experiment. **(a) **Data are pooled from 3 to 11 experiments and presented as the percent of control (vehicle-treated) counts per minute (CPM) + standard error. In 11 experiments, the average CPM in vehicle-treated controls was 35,583 + 6,893. An asterisk (*) indicates a significant decrease in ^3^H-thymidine incorporation relative to vehicle controls, *p* < 0.05. **(b) **Data obtained with galangin as above were replotted to determine the IC_50 _(median inhibition concentration) (median inhibition concentration). The calculated IC_50 _was 11 μM.

Since molecular manipulation of AhR activity can affect cell proliferation [40,44], the ability of 10^-4 ^to 10^-6 ^M galangin, α-NF, and I3C to alter Hs578T cell growth was studied. At the highest dose of 10^-4 ^M, α-NF was toxic (>50% dead as measured by PI staining) and was not assessed further at that dose for its ability to inhibit proliferation. No toxicity was observed with the other compounds at any dose or with α-NF at lower doses (<3% dead by PI staining). Addition of 10^-4 ^to 10^-5 ^M galangin significantly (*p* < 0.04) reduced cell proliferation as measured by ^3^H-thymidine incorporation (Figure 3a). At 10^-6 ^M, galangin reduced ^3^H-thymidine incorporation by approximately 25%, although this reduction was not statistically significant. Overall, the IC_50 _(median inhibition concentration) of galangin under these conditions was 11 μM (Figure 3b), a result that compares favorably with concentrations of tamoxifen required to inhibit proliferation of ER^+ ^mammary tumor cells by 50% (for example, 31 μM) [67]. Consistent with previous studies in ER^+ ^cells [18,68,69], I3C significantly reduced ^3^H-thymidine incorporation at all doses tested. Interestingly, α-NF, which was shown to be a potent AhR inhibitor in this cell line (Figure 2), had no effect on Hs578T cell proliferation.

The ability of both an AhR antagonist (galangin) and an AhR agonist (I3C) to suppress cell proliferation, and the failure of a second AhR antagonist (α-NF) to affect proliferation, suggested that AhR down-regulation is either not involved or is insufficient for galangin-dependent proliferation inhibition. Since pharmacological agents such as galangin and I3C may have multiple biological activities, a second approach, transfection with an AhR-specific repressor [53], was taken to confirm that AhR down-regulation in and of itself is not sufficient to alter Hs578T cell proliferation. An evolutionarily conserved [53,70-73] AhR repressor (AhRR) specifically blocks AhR-dependent *CYP1A1 *activity by competing for the AhR binding partner ARNT and by blocking binding of this complex to recognition sequences in target genes [53,70]. Notably, AhRR derived from killifish (*F. heteroclitus*) inhibits both human and mouse AhR-dependent transactivation in an AhR-specific manner [53]. In our hands, the *F. heteroclitus AhRR *(*FhAhRR*) expression plasmid is more effective at suppressing *pGudLuc *activity in Hs578T cells than a human *AhRR *expression construct (not shown). Therefore, the *FhAhRR *construct was used to determine if inhibition of AhR activity is sufficient to suppress Hs578T cell proliferation.

**Figure 4:**
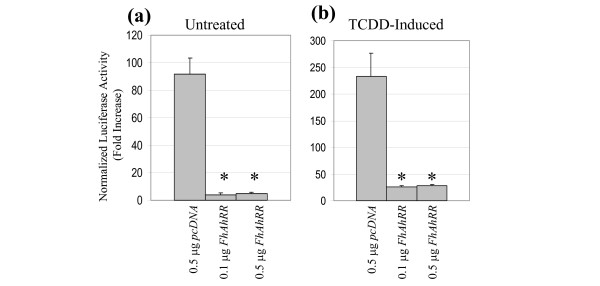
Aryl hydrocarbon receptor (AhR) repressor (*FhAhRR*) inhibits AhR-dependent *pGudLuc *reporter activity. Hs578T cells were left untransfected or were transfected with 0.5 μg/well renilla luciferase vector *phRL-TK *and 0.1 μg *pGudLuc*/well together with 0.5 μg control vector (*pcDNA*), 0.1 μg *FhAhRR*, or 0.5 μg *FhAhRR *in the **(a) **absence or **(b) **presence of 10^-9 ^M 2,3,7,8-tetrachlorodibenzo-*p*-dioxin (TCDD). Cells were harvested 18 hours later and luciferase activity assayed. Firefly luciferase activity was normalized to renilla activity in each experiment. Data pooled from six experiments are presented as the average fold increase relative to non-transfected cells + standard error. An asterisk (*) indicates a significant difference relative to pcDNA-transfected controls, *p* < 0.001.

**Figure 5:**
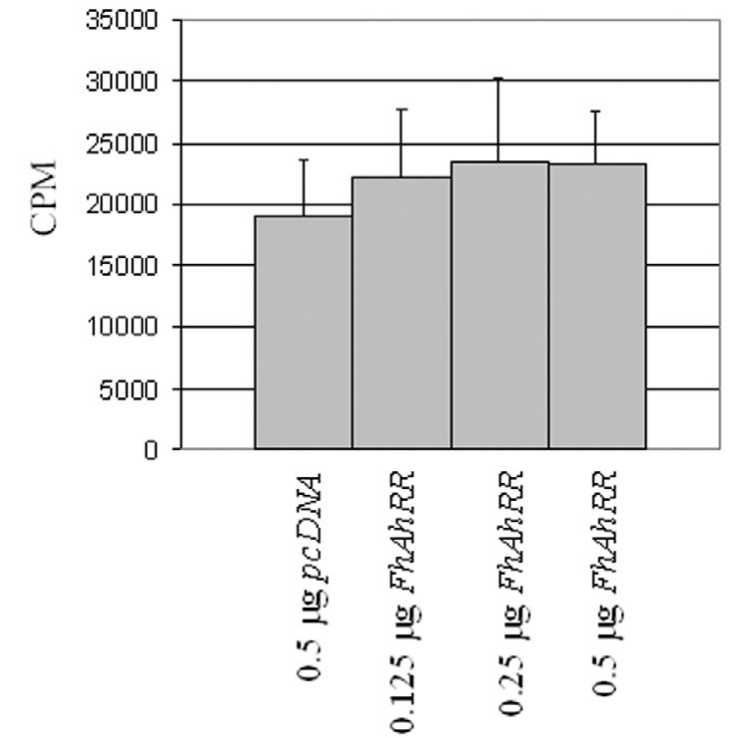
Aryl hydrocarbon receptor repressor does not inhibit proliferation of Hs578T breast cancer cells. Hs578T cells were transfected with control *pcDNA *vector or with *FhAhRR *plasmid as in Figure 4, plated in triplicate in 96-well plates, and allowed to adhere overnight before addition of ^3^H-thymidine. Cells were harvested 18 hours later and assayed for ^3^H-thymidine incorporation. Triplicates were averaged in each experiment. Data are pooled from three experiments and are presented as the mean counts per minute (CPM) + standard error. There were no statistical differences between groups.

Hs578T cells were transiently transfected with *FhAhRR *or control *pcDNA *either with *pGudLuc*, to confirm *FhAhRR *activity, or without *pGudLuc *to evaluate cell proliferation. Transfection with *FhAhRR *significantly reduced both the constitutive (Figure 4a) and the TCDD-inducible (Figure 4b) *pGudLuc *reporter activity in transfected Hs578T cells, confirming the potent inhibitory activity of ectopically expressed AhRR. However, *FhAhRR *transfection had no effect on ^3^H-thymidine incorporation (Figure 5). These results demonstrate that AhR repression is not sufficient to effect inhibition of proliferation in this cell line. It is concluded that galangin's ability to inhibit cell proliferation either doesn't involve the AhR or is mediated by AhR suppression together with other activities.

### Galangin blocks G_0_/G_1 _to S transition

**Figure 6:**
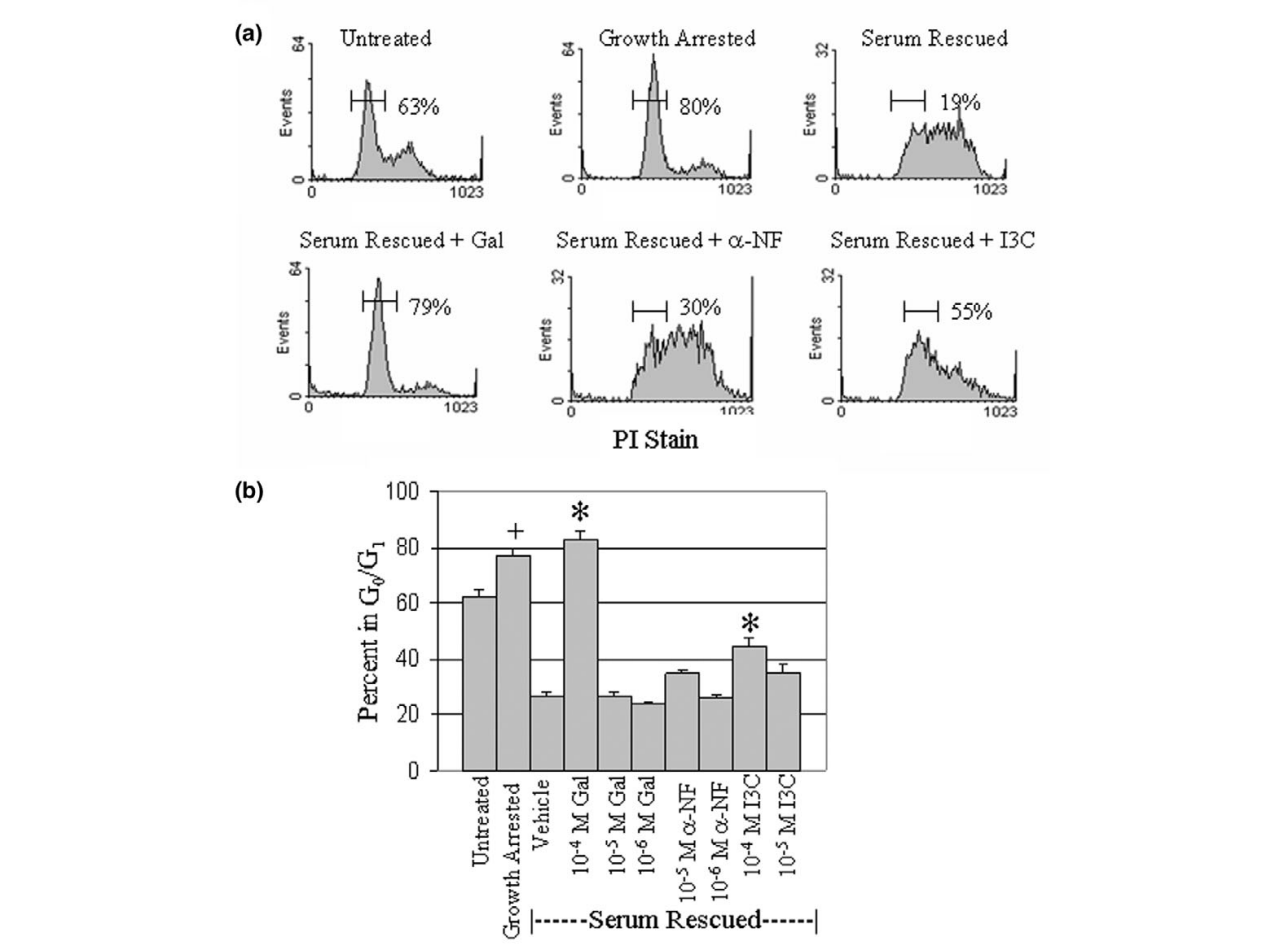
Galangin and indole 3-carbinol (I3C) block Hs578T cells progression from G_0_/G_1 _into cell cycle. Hs578T cells were synchronized by serum deprivation for 48 h followed by rescue with 10% serum. As indicated, 10^-4 ^to 10^-6 ^M galangin (Gal), 10^-5^-0^-6 ^M α-naphthoflavone (α-NF), or 10^-4 ^M I3C were added to triplicate wells at the time of serum rescue. Cells were harvested 24 hours later and assayed for DNA content by propidium iodide (PI) staining and flow cytometry. Data from triplicate wells were averaged in each experiment. **(a) **Flow cytometry histograms from one representative experiment in which 10^-4 ^M galangin, 10^-5 ^M α-NF, and 10^-4 ^M I3C were used are presented. **(b) **Data pooled from 4 to 9 experiments are presented as the mean percentage of cells in G_0_/G_1 _+ standard error. An asterisk (*) indicates a significant increase relative to serum starved, vehicle treated cultures, *p* < 0.01. A cross (+) indicates a significant increase relative to untreated cultures, *p* < 0.01.

To determine the stage(s) of cell cycle at which galangin blocks proliferation, Hs578T cells were synchronized by serum deprivation for 48 hours and then serum rescued in the presence of galangin, α-NF, or I3C. DNA content was assayed 24 hours after serum rescue by PI staining and flow cytometry. Approximately 60% of the cells growing in log phase were in the G_0_/G_1 _phase of cell growth at any given time (Figure 6a,b). Growth arrest induced by deprivation of serum significantly (*p* < 0.01) increased the number of cells in G_0_/G_1 _to approximately 80%. Addition of serum with vehicle initiated cell cycle as indicated by a decrease in the number of cells in G_0_/G_1 _to approximately 25%. However, this decrease in G_0_/G_1 _cells was not seen when serum was added in the presence of 10^-4 ^M galangin. One log less galangin had no effect on serum rescue. As expected from its failure to affect proliferation of non-synchronized cells (Figure 3), 10^-5 ^to 10^-6 ^M α-NF had no effect on the number of cells exiting G_0_/G_1 _after serum rescue (Figure 6). The highest I3C dose (10^-4 ^M) partially but significantly (*p* < 0.01) inhibited transition of cells from the G_0_/G_1 _into the S phase of cell cycle after serum rescue. Again, since I3C and its metabolites have multiple biological activities, we cannot conclude that the effect seen in H3578T cells is due to its AhR agonist activity.

Growth-arrested, serum-rescued, and flavonoid-treated cells also were assayed for apoptosis as measured by the presence of a sub G_0_/G_1 _peak as we have described [56-58]. Regardless of treatment, less than 8% of the cells were apoptotic and no differences were seen between groups at doses shown in Figure 6b (not shown). These data indicate that galangin is non-toxic and that it blocks the transition of Hs578T cells from the G_0_/G_1 _to the S phase of cell growth.

### Galangin down-regulates cyclins D3, E, and A

Cell cyclins tightly regulate the transition of cells through the phases of the cell cycle. The D cyclins are upregulated at the initiation of the cell cycle and drive cells from the G_0_/G_1 _to the S phase of growth in part through retinal blastoma protein (Rb) phosphorylation [74]. Cyclin E is upregulated by E2F released from Rb during the late phases of G_1 _and, once in complex with Cdk2, commits the cell to divide [75]. Cyclin A functions both in the S and M phases of the cell cycle [76]. Dysregulation of each of these cyclins has been associated with mammary tumorigenesis [77-79]. To determine at what level(s) galangin effects proliferation inhibition, Hs578T cells were left untreated or were treated with 10^-4 ^M galangin, 2.5 × 10^-4 ^M I3C, or 10^-5 ^M α-NF and assayed for cyclin D1, D3, E, and A expression 18 hours thereafter.

**Figure 7:**
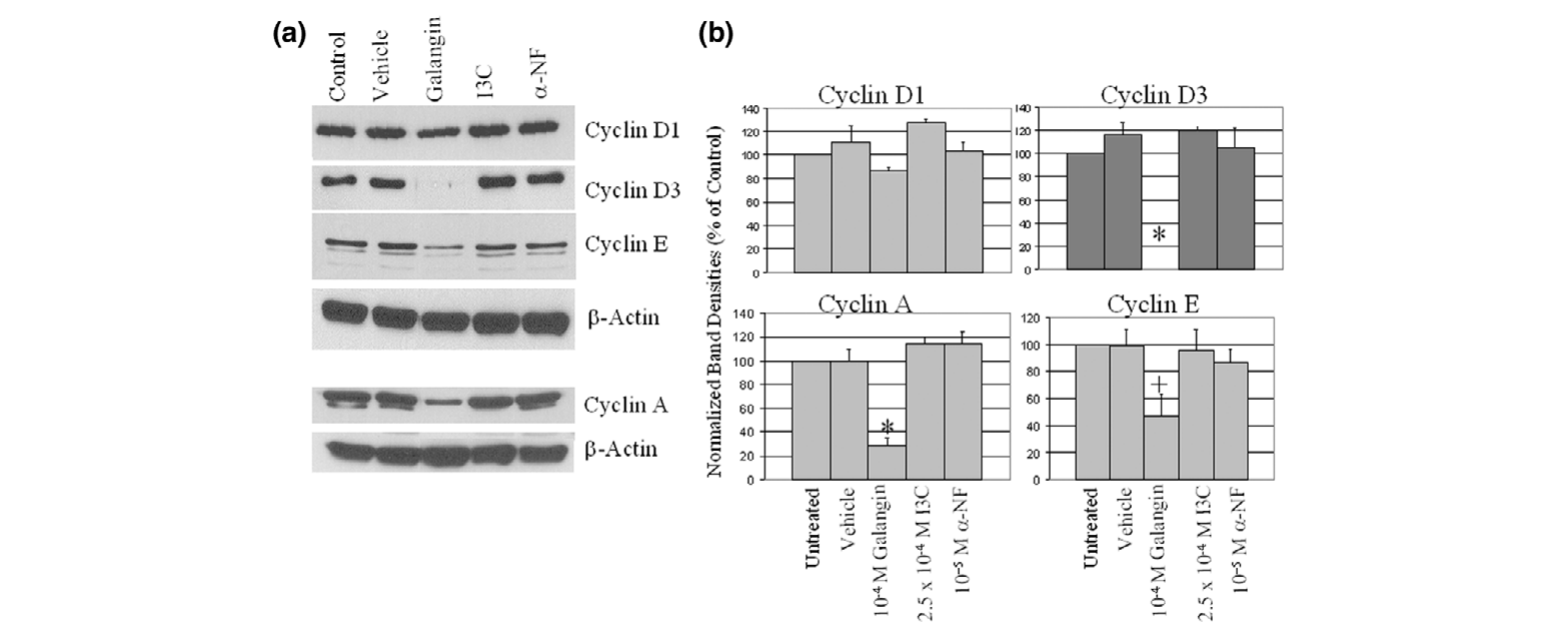
Galangin down-regulates expression of cyclins D3, A, and E. Hs578T cells were left untreated or were treated with vehicle, 10^-4 ^M galangin, 2.5 × 10^-4 ^M indole 3-carbinol (I3C), or 10^-5 ^M α-naphthoflavone (α-NF) and assayed for cyclin D1, D3, E, and A expression 18 hours thereafter by western blotting. Blots were stripped and reprobed with β-actin-specific antibody to control for sample loading. **(a) **Data from one representative experiment from a total of three experiments are presented. **(b) **Cyclin band densities were normalized with β-actin band densities and then expressed as a percentage of β-actin normalized cyclin expression in untreated cultures. Data are pooled from three experiments and expressed as the percent of control of the respective normalized band densities + standard errors. An asterisk (*) indicates a significant decrease in cyclin expression relative to vehicle controls, *p* < 0.001. A cross (+) indicates a significant decrease, *p* < 0.02.

Although galangin tended to decrease cyclin D1 expression, the data did not reach statistical significance in this series of three experiments (Figure 7a,b). However, expression of cyclin D3 was nearly undetectable in galangin-treated cells. Furthermore, galangin significantly reduced expression of cyclins A (*p* < 0.001) and E (*p* < 0.02). Since cyclins A and E function downstream of cyclin D3, these data are consistent with the cell cycle data (Figure 6) and support, but do not prove, the hypothesis that galangin blocks transition of cells from G_0_/G_1 _into S phase by profoundly down-regulating at least cyclin D3. As in previous experiments, no overt toxicity (for instance, uptake of trypan blue) was noted following galangin treatment (data not shown).

In contrast, neither I3C nor α-NF significantly affected expression of the cyclins assayed herein, even though relatively high doses were used (Figure 7a,b). The failure of I3C to inhibit expression of these cyclins, while clearly affecting cell proliferation at lower doses (Figure 3), suggests its ability to interfere with components of the cell cycle machinery not assayed here and distinct from those targeted by galangin.

## Discussion

In the search for less toxic breast cancer chemotherapeutics, many laboratories have turned their attention to naturally occurring bioflavonoids or synthetic analogues thereof. Galangin is one such polyphenolic compound that has been shown to have significant biological activity in a number of systems [7-11,13]. In our hands, galangin is a potent inhibitor of environmental chemical toxicity mediated by carcinogenic polycyclic aromatic hydrocarbons through its ability to block AhR activation [15]. In those studies, galangin inhibited AhR activation without overt toxicity to what would otherwise be considered extremely sensitive cells, that is, developing bone marrow hematopoietic cells. The lack of toxicity is supported further by the present studies, in which doses as high as 10^-4 ^M failed to induce overt cell death, as measured by trypan blue uptake, or more cryptic apoptotic death, as measured by a decrease in staining with PI in permeabilized cells.

A number of studies demonstrated that the AhR can regulate cell proliferation [42,80]. In several cases, particularly with regard to rapidly growing or transformed cells, the AhR appears to be constitutively active [44,63,81-84]. Our laboratory has shown that this phenomenon holds for rodent mammary tumors induced with prototypic AhR ligands [16]. Similarly, high levels of nuclear AhR were observed in human Hs578T cells (Figure 1) and in several other human breast cancer cell lines (for example, CAMA-1, MCF-7, and MDA MB 231; data not shown). In addition, the presence of a significant background level of *pGudLuc *reporter activity that was inhibitable with galangin, α-NF (Figure 2), or *FhAhRR *transfection (Figure 4), indicated that the AhR is constitutively active in Hs578T cells. Therefore, it is reasonable to hypothesize that AhR up-regulation is a general characteristic of mammary tumors and that it influences their growth. In support of this hypothesis, AhR inhibition through molecular manipulations, such as transfection of AhR-specific siRNA, suppresses proliferation of human hepatoma cells [40] while AhR-defective hepatoma cells grow more slowly than wild-type cells [44].

Because of these results, we had initially proposed that galangin would effect a change in mammary tumor cell proliferation through inhibition of AhR activity. Indeed, galangin was shown to both inhibit AhR activity (Figure 2) and to block cell proliferation (Figures 3 and 6). The IC_50 _of galangin (11 μM) in this system was similar to that reported for tamoxifen with ER^+ ^MCF-7 cells (31 μM)[67]. However, cell proliferation was not altered by α-NF or *FhAhRR *despite their ability to suppress AhR activity as efficiently as galangin. Therefore, it appears either that the AhR is not involved in suppressing proliferation or that AhR inhibition is not sufficient to block proliferation of this relatively advanced tumor cell line. This result does not rule out the possibility that AhR down-regulation is sufficient to alter proliferation of less aggressive mammary tumors. Indeed, recent experiments demonstrate that inhibition of AhR activity in pre-malignant, MCF-10F mammary epithelial cells through transduction with an *FhAhRR*-containing lentivirus vector profoundly inhibits proliferation (data not shown).

Furthermore, the demonstration that galangin dramatically inhibits constitutive and environmental chemical-induced *pGudLuc *activity is an important observation in and of itself. Active AhR induces transcription of *CYP1 *genes encoding enzymes that biotransform ubiquitous environmental carcinogens (for example, PAH) and putative endogenous substrates [63] into mutagenic metabolites. Consequently, non-toxic flavonoids such as galangin may be seen as potential chemopreventatives capable of blocking mutation-driven tumor initiation and/or progression through down-regulation of *CYP1 *transcription. Its ability also to block CYP1A1 enzyme activity directly [51], and to act as a free radical scavenger [2], suggests two additional levels at which galangin may restrict mutagen production or activity.

Flow cytometric studies presented herein demonstrate that galangin blocks transition of Hs578T cells from the G_0_/G_1 _into S phase of cell growth. Profound inhibition of cyclin D3 expression and the tendency to reduce cyclin D1 expression after galangin exposure are consistent with this finding since activation of cyclin D-CDK4 complexes is rate limiting in transition of cells from the G_1 _to S phase of cell growth. These observations are important since cyclin D3 plays a critical role in mammary tumorigenesis [85,86] but has not yet been specifically targeted with chemotherapeutics. Interestingly, it has been suggested that cyclin D3 preferentially promotes development of squamous carcinomas [85] and that it activates an oncogenic pathway in mammary epithelial cells that is distinct from the pathway induced by cyclin D1 [85,87]. Consequently, the preferential down-regulation of cyclin D3 by galangin may complement and increase the inhibitory effects of putative chemotherapeutics that target cyclin D1 [88] or, for that matter, other components that regulate cell cycle in tumors.

Since transcription of cyclins E and A is regulated by the D cyclins through control of Rb phosphorylation and E2F release, it is likely that cyclin D3 down-regulation is responsible for the observed decreases in cyclins E and A seen in galangin-treated cells (Figure 7). Experiments now underway are testing this and the alternative possibility, that galangin directly suppresses cyclins E and A as well as cyclin D3. In either case, the down-regulation of multiple cyclins known to be involved in mammary tumorigenesis emphasizes the potential for galangin to serve as an effective inhibitor of mammary tumor proliferation.

## Conclusion

We have described the novel finding that a naturally occurring, non-toxic bioflavonoid, galangin, effectively suppresses proliferation of an ER^- ^cell line. This proliferation inhibition is accompanied by down-regulation of cyclins D3, E, and A. While galangin inhibits the activity of the AhR, a transcription factor implicated in the initiation and growth of mammary tumors, AhR inhibition was either not required or not sufficient to suppress proliferation of this cell line. These results suggest that this bioflavonoid may represent a useful therapeutic for the treatment of ER^- ^mammary tumors and should complement the effects of therapeutics that target other dysregulated components of the cell cycle machinery.

## Abbreviations

α-NF = α-naphthoflavone; AhR = aryl hydrocarbon receptor; AhRR = aryl hydrocarbon receptor repressor; ARNT = aryl hydrocarbon receptor nuclear translocator; DMEM = Dulbecco's modified Eagle's medium; ER = estrogen receptor; FCS = fetal calf serum; I3C = indole 3-carbinol;  PAH = polycyclic aromatic hydrocarbon; PAS = Per/ARNT/Sim; PBS = phosphate-buffered saline; PI = propidium iodide; TCDD = 2,3,7,8-tetrachlorodibenzo-*p*-dioxin.

## Competing interests

The authors declare that they have no competing interests.

## Authors' contributions

TJM performed [^3^H]-thymidine incorporation experiments, cell cycle and apoptosis analyses, and western immunoblotting, participated in the experimental design and coordination of this project, and participated in writing the manuscript. XY performed transient transfections and reporter assays and western immunoblotting, participated in the experimental design and coordination of the project, and contributed to the writing of the manuscript. DHS conceived of the experimental design, performed statistical analyses, and prepared the manuscript. All authors approved the final manuscript.
